# Interventional Mitral Valve Edge‐to‐Edge Repair in Patients Above 85 Years of Age

**DOI:** 10.1002/ccd.70807

**Published:** 2026-08-12

**Authors:** Florian Jürgens, Florian Schindhelm, Mohammed Abusharekh, Jürgen Kampf, Matthias Totzeck, Fadi Al‐Rashid, Peter Lüdike, Tienush Rassaf, Amir A. Mahabadi

**Affiliations:** ^1^ Clinic of Cardiology and Vascular Medicine, West German Heart and Vascular Center Essen University Hospital Essen, University of Duisburg‐Essen Duisburg Germany; ^2^ Department of Cardiology and Intensive Care Medicine Niels‐Stensen‐Kliniken, Marienhospital Osnabrück Osnabrück Germany

**Keywords:** elderly, interventional cardiology, mitral regurgitation, M‐TEER, PASCAL, structural heart, transcatheter edge‐to‐edge therapy

## Abstract

**Background:**

Mitral valve transcatheter‐edge‐to‐edge repair (M‐TEER) is routinely performed in patients with severe mitral regurgitation (MR) at high operative risk, frequently including elderly patients. Patients ≥ 85 years, however, are frequently excluded in clinical studies.

**Objectives:**

We evaluated differences of procedural characteristics and outcomes following M‐TEER in patients < versus ≥ 85 years.

**Methods:**

We retrospectively included consecutive patients with severe MR who underwent M‐TEER with the PASCAL device between 2019 and 2024 at our center. Baseline and procedural characteristics, short‐ and mid‐term outcomes were compared, stratifying the cohort into patients < versus ≥ 85 years.

**Results:**

Three hundred seventy‐five patients with moderate to severe or severe MR were eligible; 71 aged ≥ 85 (87.0 ± 1.7 years, 57.7% male) and 304 aged < 85 (75.5 ± 10.0 years, 58.6% male) years. Patients aged ≥ 85 years more frequently had severe MR (90.1% vs. 73.8%, *p* = 0.005) and higher STS‐Score (6.4 [3.8; 9.1] vs. 3.5 [2.2; 6.5], *p* < 0.0001) at baseline. No leaflet injury, single leaflet detachment or any other device‐related complication was observed, irrespective of age. Residual MR at 90 days was comparable among age groups (Trace: 34.8% vs. 23.8%; mild: 41.3% vs. 57.3%; moderate: 23.9% vs. 18.5%; moderate‐severe: 0% vs. 0.4%, *p* = 0.56). Within 30 days, no death occurred in patients aged ≥ 85 years (one death in the < 85 years group). Median mid‐term follow‐up was 430 [164; 692] days. Overall mortality was not different for patients ≥ versus < 85 years (5.6% vs. 7.6%, *p* = 0.76).

**Conclusion:**

M‐TEER is safe in patients ≥ 85 years, with procedural outcomes and mid‐term prognosis similar to younger cohorts.

**Abbreviations**
DMR: degenerative mitral regurgitation
EROA: Effective regurgitant orifice area
FMR: functional mitral regurgitation
LVEF: Left ventricular ejection fraction
LVEDD: Left ventricular end‐diastolic diameter
LVEDV: Left ventricular end diastolic volume
LVESD: Left ventricular end‐systolic diameter
LVESV: Left ventricular end‐systolic volume
MAE: Major adverse events
MMR: mixed mitral regurgitation
MPG: Mean pressure gradient
MR: Mitral regurgitation
M‐TEER: Mitral transcatheter edge‐to‐edge repair
MV: Mitral valve
MVARC: Mitral valve academic research consortium
SLDA: single leaflet device attachments

## 1 Introduction

The utilization of mitral valve transcatheter edge‐to‐edge repair (M‐TEER) is increasingly adopted on a global scale in the context of severe mitral regurgitation (MR) associated with high surgical risk [1, 2], demonstrating significant improvements in clinical outcomes and prognosis [2, 3]. Patients of highly advanced age frequently present with more comorbidities and are subject to a markedly increased surgical risk, which makes advanced aged patient cohorts a significant target group for the transcatheter treatment of mitral regurgitation [4].

Notwithstanding, evidence is scarce in octogenarian patients, with mean ages of 72 and 70 years in the landmark COAPT and RESHAPE‐HF2 trials, respectively [2, 3]. Moreover, a considerable proportion of elderly with MR were deemed ineligible for the COAPT trial as a result of the study's stringent exclusion criteria [5, 6]. Similarly, the CLASP IID trial evaluating the PASCAL system in degenerative MR reported a mean age of approximately 81 years, further underscoring the underrepresentation of very elderly patients in randomized studies [7].

In addition to the first introduced Abbott's MitraClip, the PASCAL transcatheter valve repair system has recently become widely implemented. In this regard, the PASCAL system has consistently demonstrated improved outcomes over 2 years in both functional and degenerative MR cohorts, providing evidence that it is as safe, effective, and not inferior to the MitraClip device in symptomatic cases of significant MR that are not surgical candidates [7, 8]. Furthermore, the PASCAL system can be employed safely and with high predictability irrespective of the presence of anatomical complexity criteria and the underlying pathophysiological genesis of MR [9, 10, 11].

However, it is crucial to acknowledge the limited body of data concerning procedural characteristics, mid‐term and long‐term outcomes for M‐TEER in general, and the PASCAL System in particular, within elderly cohorts, a population that is growing in size and emerging as a significant target group for transcatheter treatment.

We therefore evaluated the short‐term and mid‐term outcomes, as well as the procedural characteristics, of patients aged ≥ 85 years who underwent M‐TEER using the Edwards PASCAL device, in comparison with a younger patient cohort receiving the same intervention. The present study also seeks to provide evidence supporting the safety and feasibility of this treatment in this vulnerable elderly population.

## 2 Methods

This single‐center retrospective study reports the results of consecutive patients undergoing successful M‐TEER for moderate‐severe (3+) or severe (4+) mitral regurgitation at the West German Heart and Vascular Center Essen between 2019 and 2024 with the use of the PASCAL transcatheter valve repair system. MR was classified according to EACVI and ESC recommendations [12]. The overall study cohort was divided according to patient age, using 85 years as the cutoff. Our study was approved by the local ethics committee (20‐9632‐BO). The study was performed in accordance to the Declaration of Helsinki and Good Clinical Practice principles. A total of 383 patients with moderate‐severe (3+) or severe (4+) MR were considered suitable for M‐TEER with the PASCAL transcatheter valve repair system; eight of those procedures were unsuccessful and have been excluded from this study, since technical success was considered the only inclusion criterion for this study. No patient was excluded from the analysis due to anatomy and underlying etiology of MR. The devices used for M‐TEER were either the PASCAL or the PASCAL ACE device (Edwards Lifesciences, Irvine, CA U.S.). The decision for an interventional approach was made by the local heart team consisting of an interventional cardiologist, a heart surgeon, and an anesthesiologist, who evaluated each patient according to the surgical risk, the anatomical complexity, the underlying pathology, and patient's comorbidities.

### 2.1 Data Collection and Echocardiographic Assessment

At baseline, we documented data regarding comorbidities, medication, laboratory results, as well as previous interventions, surgeries, or other device implantations. All patients suitable for M‐TEER received echocardiography at baseline, at discharge, after 3 months, and at mid‐term follow‐up in a multiparametric approach according to the latest ESC guideline recommendations [12], including qualitative parameters (MV morphology, color flow MR jet), semi‐quantitative parameters (Vena Contracta), quantitative parameters (EROA/Regurgitation volume), as well as structural parameters (LV and LA size). The severity of MR was graded as follows: None/trace (0), mild (1+), moderate (2+), moderate‐to‐severe (3+), and severe (4+). Additionally, MR etiology was classified as functional (FMR), degenerative (DMR), or mixed (MMR), and the anatomical complexity of each valve was assessed according to the Hausleiter criteria before implantation [13].

### 2.2 Procedure

All M‐TEERs were carried out with the use of transesophageal echocardiographic and fluoroscopic guidance and were performed either in general anesthesia or in deep sedation [14]. After the procedure, all patients were monitored at the intermediate or intensive care unit until the next day and received transthoracic echocardiography before discharge. Additionally, procedural data regarding the type of device used, procedure length and fluoroscopy duration was collected.

### 2.3 Study Endpoints

We specifically evaluated the intrahospital, 30 day, and mid‐term mortality in all patients. Further endpoints included reduction in MR, freedom from MAE, and improved NYHA functional class after 90 days, and in a mid‐term follow‐up. Safety endpoints were based on MVARC [15] and included the need for unplanned cardiac surgery, specific device‐related complications like single leaflet device attachments (SLDA), device embolization, endocarditis, LVAD implantation, and other device‐related requiring cardiac surgery. Efficacy endpoints included procedural success, based on MVARC as follows: (A) device success proving a reduction in MR grade to an optimal or acceptable level at discharge; (B) absence of MAE at discharge including cardiovascular mortality, stroke, myocardial infarction, severe life‐threatening bleeding requiring transfusions and catecholamines, severe hypotension requiring inotropic support, new onset of arrhythmias requiring permanent pacemaker implantation, acute kidney injury and additionally, arrhythmias requiring CPR [16].

### 2.4 Statistical Analysis

Continuous variables were reported as mean ± standard deviation or median (1st and 3rd quartile) and were compared (< vs. ≥ 85 years) using the *t*‐test or Wilcoxon test. Binary random variables were reported as *n* (%) and compared using the chi‐square test. For other categorical variables we report number and ratio of each expression and test for equal distributions using an ANOVA. Kaplan‐Meier analysis was performed describing the mid‐term mortality with differences among groups compared with the log‐rank test. All computations were performed using R, version 4.3.2.

## 3 Results

### 3.1 Selected Patients

Between 2019 and 2024, 375 patients with moderate‐severe (3+) or severe (4+) MR were successfully treated with M‐TEER using the PASCAL transcatheter valve repair system (Mean age: 77.6 ± 10.1 years, range 26–91 years, 58.4% male). Of those, 71 patients were aged ≥ 85 years (87.0 ± 1.7 years; 57.7% male), and 304 were aged < 85 years (mean 75.5 SD: 10; male 58.6%). Detailed baseline characteristics are summarized in Table 1.

**TABLE 1:** Baseline characteristics.

	Overall	≥ 85 years of age	< 85 years of age	*p*‐value
Age, years	77.6 ± 10.1	87 ± 1.72	75.5 ± 9.96	< 0.0001
Male sex, m, *n* (%) e	219 (58.4%)	41 (57.7%)	(58.6%)	1
BMI (in kg/m^2^)	25.7 ± 8.03	24.4 ± 2.93	26 ± 8.79	0.009
NT‐proBNP, pg/mL	2607 (1159; 5731)	3365 (1681; 5367)	2470 (1124; 5753)	0.230
Creatinine, mg/dL	1.25 (1.02; 1.6)	1.31 (1.1; 1.62)	1.23 (1; 1.58)	0.053
Hemoglobin, g/dL	12.5 (11.3; 13.9)	12.2 (10.9; 13.5)	12.6 (11.4; 14)	0.051
GFR < 30 (mL/min/1.73qm), *n* (%)	55 (14.7%)	15 (21.1%)	40 (13.2%)	0.128
GFR < 60 (mL/min/1.73qm), *n* (%)	235 (63.3%)	53 (76.8%)	182 (60.3%)	0.015
Atrial fibrillation, *n* (%)	264 (70.4%)	48 (67.6%)	216 (71.1%)	0.668
COPD, *n* (%)	37 (9.87%)	4 (5.63%)	33 (10.9%)	0.268
Coronary artery disease, *n* (%)	227 (60.5%)	46 (64.8%)	181 (59.5%)	0.497
Prior myocardial infarction, *n* (%)	54 (14.5%)	8 (11.3%)	46 (15.2%)	0.505
Previous PCI intervention, *n* (%)	153 (40.9%)	33 (46.5%)	120 (39.6%)	0.354
Peripheral Artery disease, *n* (%)	119 (31.7%)	24 (33.8%)	95 (31.2%)	0.780
Open heart surgery, *n* (%)	63 (17%)	15 (21.4%)	48 (15.9%)	0.356
HFrEF, *n* (%)	97 (25.9%)	14 (19.7%)	83 (27.4%)	0.239
HFmrEF, *n* (%)	87 (23.2%)	17 (23.9%)	70 (23%)	0.993
HFpEF, *n* (%)	142 (37.9%)	31 (43.7%)	111 (36.5%)	0.326
Diabetes mellitus, *n* (%)	74 (19.7%)	11 (15.5%)	63 (20.7%)	0.406
Arterial hypertension, *n* (%)	308 (82.1%)	63 (88.7%)	245 (80.6%)	0.150
Chronic Kidney disease, *n* (%)	157 (41.9%)	32 (45.1%)	125 (41.1%)	0.635
Stroke, *n* (%)	38 (10.2%)	2 (2.82%)	36 (11.9%)	0.040
KCCQ‐Score	54.6 ± 18.8	53.2 ± 22.2	54.7 ± 18.6	0.869
6 min Walk Test, meter	286 ± 107	251 ± 67.4	289 ± 109	0.184
STS Score mortality and morbidity	14.1 (10.4; 20.3)	15.6 (11.9; 21.8)	13.9 (9.92; 19.7)	0.019
STS Score for mortality	4.2 (2.33; 7.14)	6.41 (3.75; 9.08)	3.51 (2.21; 6.45)	< 0.0001
Functional MR, *n* (%)	114 (30.4%)	21 (29.6%)	93 (30.6%)	0.981
Degenerative MR, *n* (%)	127 (33.9%)	34 (47.9%)	93 (30.6%)	0.008
Mixed MR, *n* (%)	134 (35.7%)	16 (22.5%)	118 (38.8%)	0.015
**Baseline echocardiography measures**				
MR‐Grade	3 + : 86 (22.9%) 4 + : 289 (77.1%)	3 + : 7 71 (9.9%) 4 + : 64 (90.1%)	3 + : 79 (26%) 4 + : 225 (74%)	0.00353
RV, mL	63 (47.2; 85)	68.5 (49.2; 112)	61.5 (47; 84)	0.137
EROA, cm^2^	0.41 (0.3; 0.55)	0.435 (0.31; 0.625)	0.4 (0.3; 0.53)	0.309
Vena contracta, mm	6.49 ± 1.8	6.88 ± 2.16	6.4 ± 1.7	0.0895
LVEF, %	50 (38; 58)	52 (42.2; 58.3)	49 (36.2; 58)	0.120
LVEDD, mm	54.6 ± 12.3	52.6 ± 10.6	55 ± 12.6	0.106
LVESD, mm	43.2 ± 13.5	39.8 ± 10)	44 ± 14.1	0.022
LVEDV, mL	117 (88.1; 164)	106 (86.5; 152)	120 (88.3; 166)	0.113
LVESV, mL	54.9 (36.6; 92.1)	48 (37; 80)	57.2 (35.8; 95.6)	0.149
MPG, mmHg	2 (1; 3)	2 (1.4; 3)	2 (1; 2.3)	0.010
Mitral Valve Area (cm^2^)	4.4 ± 1.74	4.48 ± 1.46	4.38 ± 1.79	0.645
Systolic pulmonary artery pressure, mmHg	44.7 ± 14	48.4 ± 15.2	43.8 ± 13.6	0.026
TAPSE, mm	18.3 ± 5.7	18.8 ± 5.68	18.2 ± 5.7	0.472
LA Volume Index, mL/m^2^	57 (45.7; 76)	64.2 (50.5; 88.8)	56 (45.3; 73.8)	0.069
**Baseline medication**				
ACE inhibitors/ARB/ARNI, *n* (%)	191 (50.9%)	40 (56.3%)	151 (49.7%)	0.379
Betablocker, *n* (%)	267 (71.2%)	44 (62%)	223 (73.4%)	0.078
Loop diuretics, *n* (%)	213 (57%)	40 (56.3%)	173 (57.1%)	1
Thiazid diuretics, *n* (%)	34 (9.07%)	6 (8.45%)	28 (9.21%)	1
Aldosterone‐antagonist, *n* (%)	174 (46.4%)	30 (42.3%)	144 (47.4%)	0.518
Anticoalgution, *n* (%)	164 (43.7%)	34 (47.9%)	130 (42.8%)	0.515
SGLT2 Inhibitor, *n* (%)	109 (29.1%)	18 (25.4%)	91 (30%)	0.525

Eight patients have been excluded from the investigation due to unsuccessful intervention. No age‐related difference was found in these eight excluded patients (*p* = 0.997) (Supporting Information S1: Table 1). In five of those cases M‐TEER led to severe mitral stenosis, in one case maneuvering of the wire was not possible because of severe vessel kinking, in one case the device exerted too much tension on the leaflet, and in one case, a serious risk of delayed single leaflet device attachment (SLDA) was reported. Therefore, these procedures have been aborted and have been excluded since our investigation only focused on successful procedures.

### 3.2 Patients Characteristics and Baseline Echocardiography

The majority of patients had atrial fibrillation, coronary artery disease, and arterial hypertension, irrespective of age group with a high frequency of other comorbidities in both groups. At baseline, patients aged ≥ 85 years presented with slightly lower BMI (24.4 ± 2.9 vs. 26 ± 8.8, *p* = 0.009), while history of stroke was more common in the younger cohort (2.8% vs. 11.9%, *p* = 0.040). Severe renal impairment (GFR < 30 mL/min/1.73 m^2^) was more frequent in the older group, though not statistically significant (21.1% vs. 13.2%, *p* = 0.128), whereas moderate renal impairment (GFR < 60 ml/min/1.73 m^2^) was significantly more prevalent among patients aged ≥ 85 years (76.8% vs. 60.3%, *p* = 0.015). Severe (4+) MR at baseline was more frequently present in patients aged ≥ 85 years (90.1% vs. 74% for older vs. younger patients, *p* = 0.0035). Similarly, patients aged ≥ 85 years also had higher STS‐Scores for morbidity (6.4 [3.8; 9.1] vs. 3.5 [2.2; 6.5], *p* < 0.0001), and higher STS‐Scores for mortality and morbidity (15.6 [11.9; 21.8] vs. 13.9 [9.92; 19.7], *p* = 0.0191), and a higher rate of pulmonary hypertension (76.1% vs. 57.9%; *p* = 0.0072). MR etiologies were also different among age groups, with degenerative MR (DMR) being more prevalent in patients aged ≥ 85 years, while mixed MR (MMR) was more prevalent in patients < 85 years (DMR: 47.9% vs. 30.6%; *p* = 0.008, FMR: 29.6% vs. 30,6%, *p* = 0.981; MMR: 22.5% vs. 38.8%, *p* = 0.014). Detailed echocardiographic baseline data are also summarized in Table 1. Regarding the anatomical complexity criteria, flail width > 15 mm and/or flail gap > 10 mm were more frequently prevalent in patients ≥ 85 years of age (16.9% vs. 7.6%, *p* = 0.027), whereas all other complexity criteria did not differ among age groups (Table 2).

**TABLE 2:** Anatomical complexity criteria according to Hausleiter [13].

	Overall	≥ 85 years of age	< 85 years of age	*p*‐value
≥ 2 independent Jets, *n* (%)	66 (17.6%)	11 (15.5%)	55 (18.1%)	0.730
MVO < 4 cm^2^, *n* (%)	135 (40.3%)	24 (41.4%)	111 (40.1%)	0.970
Bileaflet/Multi Scallop prolapse involvement, *n* (%)	64 (17.2%)	18 (25.4%)	46 (15.2%)	0.063
Significant Jet in the commissural area, *n* (%)	64 (17.1%)	17 (23.9%)	47 (15.5%)	0.125
Flail width > 15 mm and/or flail gap > 10 mm, *n* (%)	35 (9.33%)	12 (16.9%)	23 (7.57%)	0.027
Other^a^, *n* (%)	64 (17.1%)	12 (16.9%)	52 (17.1%)	1

^a^ Other complexity criteria included significant cleft or perforation in grasping area, moderate to severe calcification in the grasping area, leaflet mobility length < 8 mm, and history of endocarditis and significant tissue defects in the leaflet.

### 3.3 Procedural Characteristics

The average procedure length overall was 91.1 ± 48.4 min and did not differ among age groups (89.4 ± 45.9 vs. 91.5 ± 49, *p* = 0.73). Mean fluoroscopy duration overall was 13.4 (Q1: 9.62; Q3: 19.1) minutes (≥ 85 years: 11.7 [9.05; 17.1) vs. < 85 years: 14.1[9.88; 19.5]). The utilization of the PASCAL P10 and the PASCAL ACE was similar among age groups (P10 62.0% vs. 64.1%; ACE 38.0% vs. 33.6%; P10 and ACE 0% vs. 2.3%). Likewise, the number of devices implanted did not differ between age groups (one device: 76.1% vs. 69.4%; two devices: 21.1% vs. 30.3%, three devices: 2.8% vs. 0.3%, *p* = 0.5). Detailed procedural characteristics are depicted in Supporting Information S1: Table 1.

Irrespective of age, no case of leaflet injury, SLDA, chordal entrapment, or any other device‐related complication was observed. Also, no case of unplanned cardiac surgery, endocarditis, or need for new LVAD implantation has occurred. Freedom of MAE (according to MVARC) was achieved in 95.5% of all patients (≥ 85 years: 94.4%; < 85 years: 95.7%), showing no significant difference between both age groups (*p* = 0.86). Specific MAE types, including bleeding and renal complications, were comparable between groups (all *p* > 0.05; Supporting Information S1: Table 2).

### 3.4 Echocardiographic Results and Outcomes at Discharge 3 Month and Mid‐Term Follow‐Up

The degree of residual MR was slightly better in the < 85 group at discharge. This difference was no longer observed at 3‐months follow‐up (Central Illustration 1, 1). Likewise, NYHA‐class improved in both groups from baseline to 3‐months and at mid‐term follow‐up, again with no differences in NYHA class among patients aged < versus ≥ 85 years (Figure 1, 1, Table 3).

**Central_Illustration 1:**
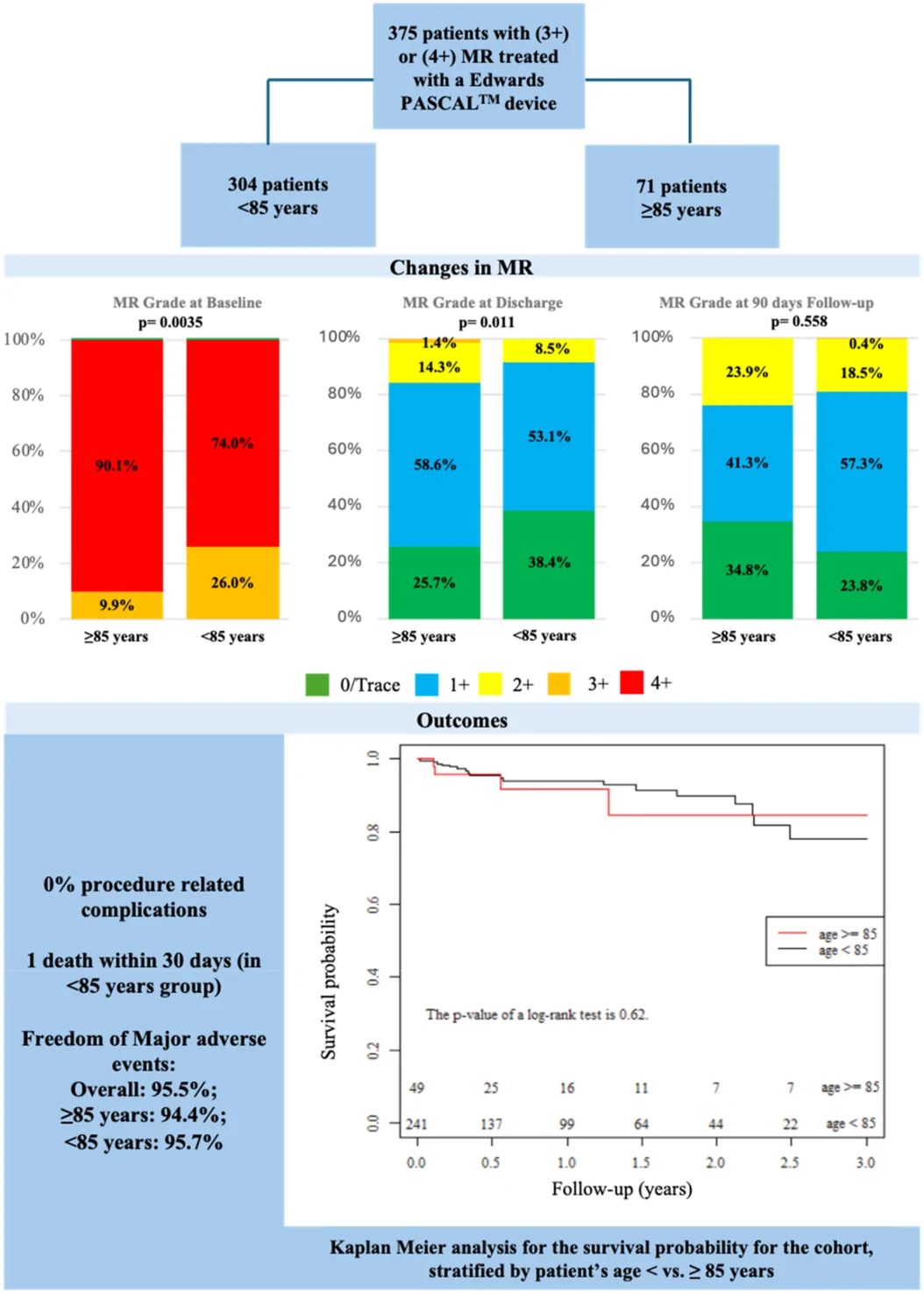
Reduction of MR and outcomes of mitral valve transcatheter edge‐to‐edge repair using the PASCAL device in patients ≥ versus < 85 years of age. [Color figure can be viewed at wileyonlinelibrary.com]

**FIGURE 1:**
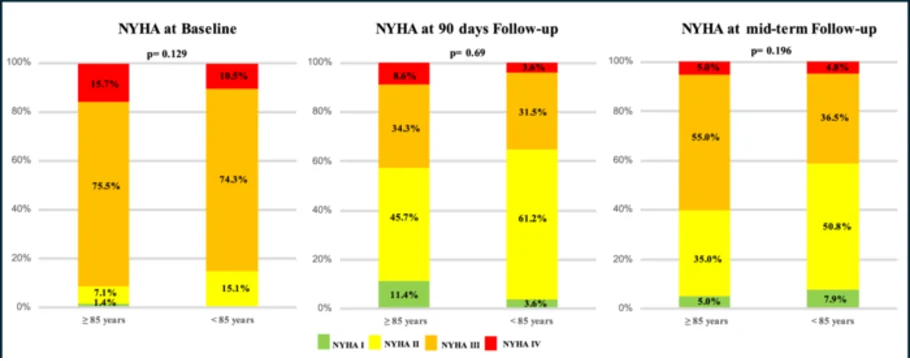
NYHA functional class at baseline, 90 days and mid‐term follow‐up (median 430 [164; 692] days), stratified for patients aged ≥ versus < 85 years. [Color figure can be viewed at wileyonlinelibrary.com]

**TABLE 3:** Echocardiographic results at discharge and 90 days.

	Overall	≥ 85 years of age	< 85 years of age	*p*‐value
**Echocardiography imaging at discharge**				
MR‐Grade	0: 131 (36%) 1 + : 197 (54.1%) 2 + : 35 (9.62%) 3 + : 1 (0.275%)	0: 18 (25.7%) 1 + : 41 (58.6%) 2 + : 10 (14.3%) 3 + : 1 (1.43%)	0: 113 (38.4%) 1 + : 156 (53.1%) 2 + : 25 (8.5%)	0.0111
EROA, cm^2^	0.194 ± 0.108	0.2 ± 0.0752	0.193 ± 0.113	0.865
Regurgitation Volume, mL	29.3 ± 20.3	28.8 ± 13	29.4 ± 21.4	0.938
Systolic pulmonary artery pressure, mmHg	41.4 ± 11.7	41.3 ± 9.13	41.5 ± 12.3	0.915
MPG, mmHg	3 (2; 4)	3 (3; 4.25)	3 (2; 4)	0.006
HR at MPG measurement, bpm	75.4 ± 37	84.4 ± 77.8	73.3 ± 16.5	0.261
Vena Contracta, mm	2.94 ± 1.04	3.02 ± 1.22	2.92 ± 0.991	0.54
**Echocardiography imaging at 90 days**				
MR‐Grade	0: 70 (25.6%) 1 + : 149 (54.6%) 2 + : 53 (19.4%) 3 + : 1 (0.366%)	0: 16 (34.8%) 1 + : 19 (41.3%) 2 + : 11 (23.9%)	0: 54 (23.8%) 1 + : 130 (57.3%) 2 + : 42 (18.5%) 3 + : 1 (0.441%)	0.558
EROA, cm^2^	0.222 ± 0.118	0.25 ± 0.0992	0.216 ± 0.122	0.480
Regurgitation Volume, mL	39.1 ± 22	50.7 ± 23.2	36.7 ± 21.4	0.214
Systolic pulmonary artery pressure, mmHg	39.9 ± 11.1	41 ± 11.2	39.6 ± 11.1	0.475
MPG, mmHg	3 (2; 4.9)	4 (3; 4)	3 (2; 5)	0.649
HR at MPG measurement, bpm	72.1 ± 19.6	70.1 ± 18.6	72.5 ± 19.8	0.460
Vena Contracta, mm	3.18 ± 1.28	3.4 ± 1.37	3.14 ± 1.26	0.266
From Implantation to first FU, days	104 (83.5; 134)	105 (80; 125)	103 (84.2; 134)	0.650

Within 30 days post‐procedure, no deaths have occurred in patients ≥ 85 years, and only one death was reported in the younger age group due to non‐procedure‐related comorbidity. The median mid‐term follow‐up was 430 [164; 692] days and the overall mortality was not different for patients aged ≥ versus < 85 years (5.6% vs. 7.6%, *p* = 0.76, Central Illustration 1, 1). The time span after successful device implantation until discharge was different between the groups with 5 days in cohort ≥ 85 years [4; 7] and 4 days in cohort < 85 years [3; 6], (*p* = 0.0037).

## 4 Discussion

This study presents a single‐center experience of M‐TEER utilizing the PASCAL transcatheter valve repair system, with a focus on safety and outcomes in patients aged < versus ≥ 85 years. The principal findings are as follows: (I) across the entire cohort of 375 patients, no incidences of unplanned cardiac surgery, device embolization, endocarditis, need for left ventricular assist device (LVAD) implantation, single‐leaflet device attachment (SLDA), chordal entrapment, leaflet injury, or other device‐related complications were observed, irrespective of age. Within 30 days post‐procedure, only one death occurred, in a patient aged < 85 years, attributable to a non‐procedural comorbidity, underscoring the high safety profile of the PASCAL transcatheter valve repair system, even among patients of very advanced age. (II) Reduction of MR was achieved in both age groups; although results at discharge slightly favored the younger cohort, the clinical benefit was sustained and comparable across cohorts at the 90‐day follow‐up. (III) Improvement in NYHA functional class was also similar regardless of age. (IV) Mid‐term mortality rates were very low in the elderly cohort, suggesting durable clinical benefit following M‐TEER. In the overall study cohort, M‐TEER employing the PASCAL transcatheter valve repair system was highly feasible and exhibited a favorable safety and efficacy profile in patients aged ≥ 85 years, with procedural success, symptomatic improvement, and mid‐term outcomes comparable to those observed in younger cohorts.

### 4.1 Comparison With Prior Literature

Evidence from large‐scale meta‐analysis indicates a pronounced age‑dependent increase in moderate‑to‑severe mitral regurgitation, identifying advanced chronological age as a key determinant in MR burden [17]. Transcatheter edge‑to‑edge repair (M‑TEER) has become a pivotal treatment modality for severe MR in elderly patients with elevated or prohibitive surgical risk; nonetheless, real‑world data in octogenarian and nonagenarian populations remain strikingly sparse—applicable to MitraClip, and even more so with the PASCAL transcatheter valve repair system [17, 18, 19]. The findings of our study are corroborated by prior investigations evaluating the safety and efficacy of M‐TEER—predominantly using the MitraClip—in elderly cohorts. In a single‐center cohort study, Lee et al. investigated 19 octogenarian patients (mean age 84.2 ± 4.0 years) and reported favorable procedural success and 1‑year outcomes [20]. Although methodologically constrained by a limited sample size and short follow‐up period, the study reinforces the procedural safety of M‑TEER in advanced‐age patients and aligns with the results of the present analysis. Likewise, a previous study in very elderly individuals undergoing MitraClip reported comparable acute procedural efficacy among octogenarians and nonagenarians relative to younger cohorts. However, the study lacked granular imaging data, pathophysiological insights into the underlying mechanisms of MR, and long‐term follow‐up [21]. In contrast, our study includes a substantially larger, well‐characterized cohort aged ≥ 85 years (*n* = 71), including eight nonagenarians, a notably high‐risk subgroup, and is strengthened by comprehensive echocardiographic assessment and extended longitudinal follow‐up, thereby supporting the safety and feasibility of the PASCAL transcatheter valve repair system in this understudied population.

### 4.2 Advanced Age and Clinical Implications

The convergence of advanced age and frailty has emerged as an area of growing interest within the M‐TEER literature. Frailty, a multidimensional clinical syndrome resulting from progressive deterioration across multiple physiologic systems [22], becomes increasingly prevalent with advancing age and is recognized as a significant predictor of adverse cardiovascular outcomes in older populations [23]. In our cohort, Baseline characteristics were largely comparable across age groups, consistent with the modest differences observed in STS scores. Although patients ≥ 85 years exhibited lower BMI and a higher prevalence of moderate renal dysfunction (GFR < 60 mL/min/1.73 m^2^), the incidence of prior stroke was significantly higher in the younger cohort. Severe renal impairment (GFR < 30 mL/min/1.73 m^2^) was numerically more frequent in the elderly but did not reach statistical significance, a threshold known to influence procedural risk scores. Crucially, these baseline disparities did not translate into measurable differences in procedural success or early outcomes, suggesting that the clinical efficacy of PASCAL M‐TEER remains consistent across the spectrum of age‐related comorbidity profiles. A contemporary prospective trial evaluating frail versus non‐frail patients subjected to M‐TEER found no significant differences in interventional success, early device performance, or acute procedural complications. While frail patients were characterized by advanced age and impaired renal function, procedural outcomes remained comparable across groups [24]. Notably, the study documented extended hospitalization in frail patients, in concordance with our observation of significantly prolonged intervals from procedure to discharge among patients aged ≥ 85 years (*p* = 0.000375).

The OCEAN‐Mitral registry demonstrated high procedural success in M‐TEER, irrespective of frailty—a result that aligns with our study, as we report full procedural success and 30‐day survival in patients aged ≥ 85 years, along with consistent MR severity reduction at discharge without significant age‐related disparities [25]. Notably, the registry reported significantly higher STS scores for mitral valve repair in patients with advanced frailty, consistent with the elevated STS scores observed in our patients aged ≥ 85 years [26]. However, our cohort remained within a moderate risk range, suggesting a comparatively favorable baseline risk profile despite advanced chronological age.

In contrast, conflicting evidence has been reported and warrants careful consideration. A U.S. National Inpatient Sample (NIS) analysis indicated that frail elderly patients undergoing M‐TEER experienced significantly higher rates of in‐hospital mortality, acute respiratory compromise, and sepsis, along with prolonged hospitalization and increased consumption of healthcare resources [26]. A more detailed examination of this analysis suggests that chronological age alone may not be the primary determinant of procedural risk; the age difference between frail and non‐frail cohorts was minimal and not statistically significant (*p* = 0.76), whereas comorbid conditions such as atrial fibrillation and liver disease were significantly more prevalent in the frail population [27]. In line with this, our data show that elderly patients (≥ 85 years) with a comorbidity profile comparable to younger patients exhibited similar procedural outcomes, despite only moderately increased STS scores within an intermediate risk range (3%–6%).

Similar findings have been observed in other studies, linking frailty to increased susceptibility to complications and extended postprocedural recovery in this older population [27]. Collectively, these data support the concept that biological age, as reflected by comorbidity burden and risk stratification scores, appears to exert a greater influence on outcomes than chronological age alone. These observations emphasize the necessity of implementing individualized treatment strategies that incorporate frailty assessment, prehabilitation and preventive interventions in this clinically vulnerable cohort.

The PASCAL transcatheter valve repair system incorporates several design aspects that differ from the MitraClip, including a more flexible delivery system, a central spacer with broader paddles designed to minimize leaflet stress (in case of the PASCAL P10 system), the possibility for independent leaflet capture (also available in the Gen4 MitraClip), and a different architecture of the retention elements [28]. Clinical data have demonstrated its procedural safety, technical feasibility, and sustained efficacy over a 1‐year follow‐up period, even in a broadly inclusive cohort characterized by heterogeneous mitral valve anatomy and diverse underlying etiologies, irrespective of anatomical or pathophysiological variability [9, 10, 11]. However, the evidence base remains limited in older patients with complex comorbid profiles, highlighting the need for dedicated evaluation of its clinical performance and therapeutic utility specifically within this challenging subgroup.

### 4.3 Performance of the Pascal System in Complex Anatomy

Our results demonstrate that the PASCAL repair system achieves high procedural success with excellent safety and favorable outcomes, even in degenerative MR with anatomically complex features. This is particularly noteworthy when compared with the CLASP IID trial, the only randomized controlled trial investigating the PASCAL system in DMR patients, which enrolled a selected cohort with non‐complex or mildly complex mitral valve anatomy [7, 29]. In contrast, our study included a considerable proportion of patients with complex anatomical features according to the established Hausleiter criteria [13], including multiple regurgitant jets (17.6%), bileaflet or multi‐scallop prolapse (17.2%), commissural involvement (17.1%), and large flail gaps (> 10 mm) or widths (> 15 mm) (9.3%). Despite this increased anatomical complexity, procedural success and early outcomes remained favorable, demonstrating the feasibility and safety of the PASCAL system in this challenging population. While previous studies focused on non‐complex DMR, our findings extend the evidence to anatomically complex cases, supporting the robust performance of the PASCAL system across a broad spectrum of mitral valve anatomy, as has also been described in a prospectively enrolled study from our center [9].

### 4.4 Future Directions

Given the global demographic shift toward an expanding population of very elderly individuals and the well‐established age‐related increase in MR prevalence, the adoption of less invasive treatment options like M‐TEER in patients aged ≥ 85 years is anticipated to rise significantly [30, 31]. In this context, our study provides a meaningful contribution to the evolving literature by presenting comprehensive procedural and follow‐up data in this advanced‐age cohort [32, 33, 34]. Our findings affirm the procedural safety, clinical efficacy, and mid‐ to long‐term durability of M‐TEER performed using the PASCAL transcatheter valve repair system in patients aged ≥ 85 years, including nonagenarians. These results underscore the importance of precision medicine‐based, age‐sensitive therapeutic decision‐making in structural heart interventions, particularly as treatment expands to encompass increasingly medically complex and older populations. Future multicenter prospective studies and registries are needed to further validate these findings and refine patient selection in elderly and frail populations.

## 5 Limitations

Our study, conducted at the West German Heart and Vascular Center Essen between 2019 and 2024 had some limitations: First, this is a single‐center study, and therefore, the study was limited by the sample size as only 71 could be assigned to the cohort of patients aged ≥ 85. In addition, the retrospective design without strict standardization for baseline‐ and follow‐up examinations represents a limitation of our study. To provide even better and more accurate results, a multicenter analysis of patients aged ≥ 85 years who are treated with the PASCAL transcatheter valve repair system or any other device suitable for M‐TTER may be needed to confirm our results. Furthermore, our study was conducted in a high‐volume institution, our results may not be generalizable to centers with lower patient volume and less experience.

## 6 Conclusion

This retrospective single‐center analysis showed that transcatheter edge‐to‐edge repair for mitral valve regurgitation can be safely performed in patients above the age of 85 years. Procedural outcomes, improvement in NYHA functional class as well as mid‐term mortality were comparable among patients < versus ≥ 85 years of age. Consequently, chronological age alone should be considered of secondary importance; instead, clinical decision‐making should be primarily guided by comorbidity burden, frailty status, and individualized risk stratification.

## Conflicts of Interest

Florian Schindhelm: received travel grants and speaker honoraria from Edwards Lifesciences. Peter Lüdike: received research grants and speaker honoraria from Edwards Lifesciences. Tienush Rassaf: received honoraria, lecture fees, and/or grant support from Edwards Lifesciences, AstraZeneca, Bayer, Novartis, Berlin Chemie, Daiicho‐Sankyo, Boehringer Ingelheim, Novo Nordisk, Cardiac Dimensions, and Pfizer, all unrelated to this work. Co‐founder of Mycor GmbH, a company focusing on AI‐based EKG‐algorithms; Amir Mahabadi: Speaker fees and/or advisory boards: Amgen, Daiichi Sankyo, Edwards, Novartis, Sanofi. Research funding: Daiichi Sankyo, Edwards, all outside the submitted work. Co‐founder of Mycor GmbH, a company focusing on AI‐based EKG‐algorithms. The other authors declare no conflicts of interest.

## Supporting information


Supporting File

## Data Availability

Research data are not shared.
