# Supplementary material for: Interventional Mitral Valve Edge‐to‐Edge Repair in Patients Above 85 Years of Age
Source: Catheter Cardiovasc Interv. 2026 Aug 12;108(4):1548–57. doi: 10.1002/ccd.70807 (PMC13667317; doi:10.1002/ccd.70807)
Supplement: Supplementary file 1 [file CCD-108-1548-s001.docx]

**Supplementary table 1**: Procedural characteristics, stratified by patients’ age

|  | **Overall** | **≥85 years of age** | **>85 years of age** | **p-Value** |
| --- | --- | --- | --- | --- |
| Number of devices, n (%) | 1: 265(70.7 %)  2: 107(28.5 %)  3: 3(0.8%) | 1:54(76.1%)  2:15(21.1%)  3: 2(2.82%) | 1:211(69.4%)  2: 92(30.3%)  3: 1(0.329%) | 0.509 |
| PASCAL10 = 1, PASCAL ACE=2, PASCAL10 AND PASCAL ACE=3, n (%) | 1: 239(63.7 %)  2: 129(34.4 %)  3: 7(1.87 %) | 1: 44(62 %)  2: 27(38 %) | 1:195(64.1%)  2:102(33.6%)  3:7(2.3%) | 0.985 |
| Procedure length, minutes | 91.1 ± 48.4 | 89.4 ± 45.9 | 91.5 ± 49 | 0.735 |
| Fluoroscopy duration, minutes | 13.4 (9.62;  19.1) | 11.7 (9.05; 17.1) | 14.1 (9.88; 19.5) | 0.150 |
| Leaflet Injury, n (%) | 0 (0 %) | 0 (0 %) | 0 (0 %) | NaN |
| SLDA, n (%) | 0 (0 %) | 0 (0 %) | 0 (0 %) | NaN |
| Chordal entrapment, n (%) | 0 (0 %) | 0 (0 %) | 0 (0 %) | NaN |
| MR reduction post-Implant release: 0=MR reduction unchanged,  1=MR reduction improved (if residual MR before),  2= recurrence of MR (if no MR before),  3= increase of residual MR,  4= development of significant new Jet, n (%) | 0: 312 (84.6 %)  1: 50 (13.6 %)  2: 2 (0.542 %)  3: 4 (1.08 %)  4: 1 (0.271 %) | 0:57(81.4%)  1: 11 (15.7%)  3: 2 (2.86%) | 0:255(85.3%) 1: 39 (13 %) 2: 2(0.669%) 3: 2(0.669%) 4: 1(0.334%) | 0.336 |
| Unsuccessful (excluded) | 8 | 1 | 7 | 0.977 |
| discharge days from procedure | 4 (3; 6) | 5 (4;7) | 4 (3;6) | 0.00375 |
| Proportion of patients with ≥2-grade MR reduction | | | | |
| MR discharge vs. MR baseline | 291 (80.2 %) | 55 (78.6 %) | 236 (80.5 %) | 0.837 |
| MR 3 months follow-up vs. MR baseline | 179 (65.8 %) | 32 (69.6 %) | 147 (65 %) | 0.675 |
| MR latest follow-up vs. MR baseline | 124 (66.3 %) | 13 (54.2 %) | 111 (68.1 %) | 0.264 |
| Proportion of patients with NYHA class III-IV | | | | |
| Baseline | 322 (85.9 %) | 64 (90.1 %) | 258 (84.9 %) | 0.338 |
| 3 months follow-up | 73 (19.5%) | 15 (21.1%) | 58 (19.1 %) | 0.821 |
| latest follow-up | 64 (17.1%) | 12 (16.9 %) | 52 (17.1 %) | 1 |

**Supplementary table 2:** Major adverse events based on MVARC, Short and mid-term mortality

|  | **Overall** | **≥85 years of age** | **>85 years of age** | **p-Value** |
| --- | --- | --- | --- | --- |
| MAE based on MVARC in total, n (%) | 17 (4.53 %) | 4 out of 71 (5.63 %) | 13 (4.28 %) | 0.859 |
| Types of Major Adverse Events | | | | |
| Major Bleeding | 3 (0.8 %) | 1 (1.41 %) | 2 (0.658 %) | 1 |
| Requirement of new Pacemaker implantation | 1 (0.267 %) | 1 (1.41 %) | 0 (0 %) | 0.427 |
| Acute Kidney injury | 5 (1.33 %) | 2 (2.82 %) | 3 (0.987 %) | 0.525 |
| Stroke | 1 (0.267 %) | 0 (0 %) | 1 (0.329 %) | 1 |
| Catecholamine requirement in case of heart failure, respiratory failure | 9 (2.4 %) | 2 (2.82 %) | 7 (2.3 %) | 1 |
| Others* | 11 (2.93 %) | 2 (2.82 %) | 9 (2.96 %) | 1 |
| Deaths | 27 (7.2 %) | 4 (5.63 %) | 23 (7.57 %) | 0.755 |
| Deaths within 90 days | 7 (1.87 %) | 2 (2.82 %) | 5 (1.64 %) | 0.865 |

*Others: including cardiogenic/ hemorrhagic shock, sepsis, iatrogenic atrial septal defect requiring intervention
